# Supplementary material for: Genome-Wide Identification and Expression Analysis of the PLATZ Transcription Factor in Tomato
Source: Plants (Basel). 2023 Jul 13;12(14):2632. doi: 10.3390/plants12142632 (PMC10384190; doi:10.3390/plants12142632)
Supplement: Supplementary file 1 [file plants-12-02632-s001.zip › plants-2419762-supplementary.pdf]

Table S1. *PLATZ* genes Identified from Whole Genomes of Heinz and Pennellii Tomatoes.

	Gene Name	Gene ID	Chromosome	Number of Amino Acids (aa)	Molecular Weight (kDa)	Theoretical pI	Aliphatic Index	Grand Average of Hydropathicity (GRAVY)	Subcellular Localization
Heinz	*SlPLATZ1*	Solyc01G002693	1	250	27.77	8.76	77.96	-0.272	nucl
	SlPLATZ2	Solyc02G001103	2	241	27.40	6.64	72.37	-0.445	nucl
	SlPLATZ3	Solyc02G000559	2	147	17.04	8.97	86.12	-0.283	nucl
	SlPLATZ4	Solyc02G000544	2	136	16.02	9.42	81.76	-0.537	cyto
	SlPLATZ5	Solyc02G000562	2	179	20.75	8.96	87.60	-0.204	nucl
	SlPLATZ6	Solyc02G000547	2	141	16.18	8.76	93.26	-0.150	cyto
	SlPLATZ7	Solyc02G000549	2	159	18.51	9.35	75.41	-0.606	cyto
	SlPLATZ8	Solyc02G000493	2	141	16.10	8.43	86.38	-0.320	cyto
	SlPLATZ9	Solyc02G000551	2	147	16.83	9.19	90.75	-0.217	golg
	SlPLATZ10	Solyc02G000554	2	198	23.36	8.61	77.27	-0.559	nucl
	SlPLATZ11	Solyc02G000552	2	124	14.69	9.02	91.13	-0.310	mito
	SlPLATZ12	Solyc02G000553	2	90	10.38	9.14	113.78	0.681	cyto
	SlPLATZ13	Solyc02G000550	2	133	15.49	8.39	87.14	-0.417	cyto
	SlPLATZ14	Solyc02G000490	2	174	19.94	7.05	83.45	-0.448	nucl
	SlPLATZ15	Solyc03G000836	3	126	14.44	9.02	82.70	-0.571	cyto
	SlPLATZ16	Solyc04G000228	4	213	24.73	9.18	78.22	-0.570	nucl
	SlPLATZ17	Solyc06G001392	6	224	25.34	9.54	66.56	-0.651	nucl
	SlPLATZ18	Solyc07G000243	7	255	29.16	8.55	69.96	-0.433	nucl
	SlPLATZ19	Solyc07G001813	7	164	18.88	6.19	77.20	-0.384	cyto
	SlPLATZ20	Solyc07G001814	7	194	22.48	9.3	66.80	-0.785	nucl
	SlPLATZ21	Solyc08G001967	8	228	25.57	9.39	64.96	-0.616	nucl
	SlPLATZ22	Solyc08G000013	8	224	25.63	9.37	80.89	-0.498	nucl
	SlPLATZ23	Solyc10G002869	10	205	22.90	9.08	78.93	-0.393	nucl
	SlPLATZ24	Solyc12G000451	12	246	28.03	8.45	73.25	-0.427	cyto
Spenn	*SpPLATZ1*	Sopen01g036730	1	250	27.78	8.67	76.40	-0.304	nucl
	SpPLATZ2	Sopen02g010840	2	159	18.43	9.33	84.59	-0.488	cyto
	SpPLATZ3	Sopen02g011530	2	159	18.59	9.58	76.67	-0.606	nucl
	SpPLATZ4	Sopen02g011590	2	159	18.51	9.62	78.49	-0.513	cyto
	SpPLATZ5	Sopen02g017650	2	241	27.52	6.74	72.37	-0.461	nucl
	SpPLATZ6	Sopen04g003230	4	204	23.71	9.11	75.00	-0.600	nucl
	SpPLATZ7	Sopen06g021440	6	225	25.44	9.54	66.27	-0.655	nucl
	SpPLATZ8	Sopen07g003380	7	255	29.16	8.55	69.96	-0.433	nucl
	SpPLATZ9	Sopen07g024560	7	186	21.41	8.96	69.68	-0.660	nucl
	SpPLATZ10	Sopen08g001120	8	224	25.55	9.44	81.74	-0.491	nucl
	SpPLATZ11	Sopen08g025390	8	248	28.00	9.49	66.01	-0.648	nucl
	SpPLATZ12	Sopen10g035200	10	205	22.89	9.08	78.44	-0.391	nucl
	SpPLATZ13	Sopen12g005480	12	246	28.03	8.45	73.25	-0.427	cyto

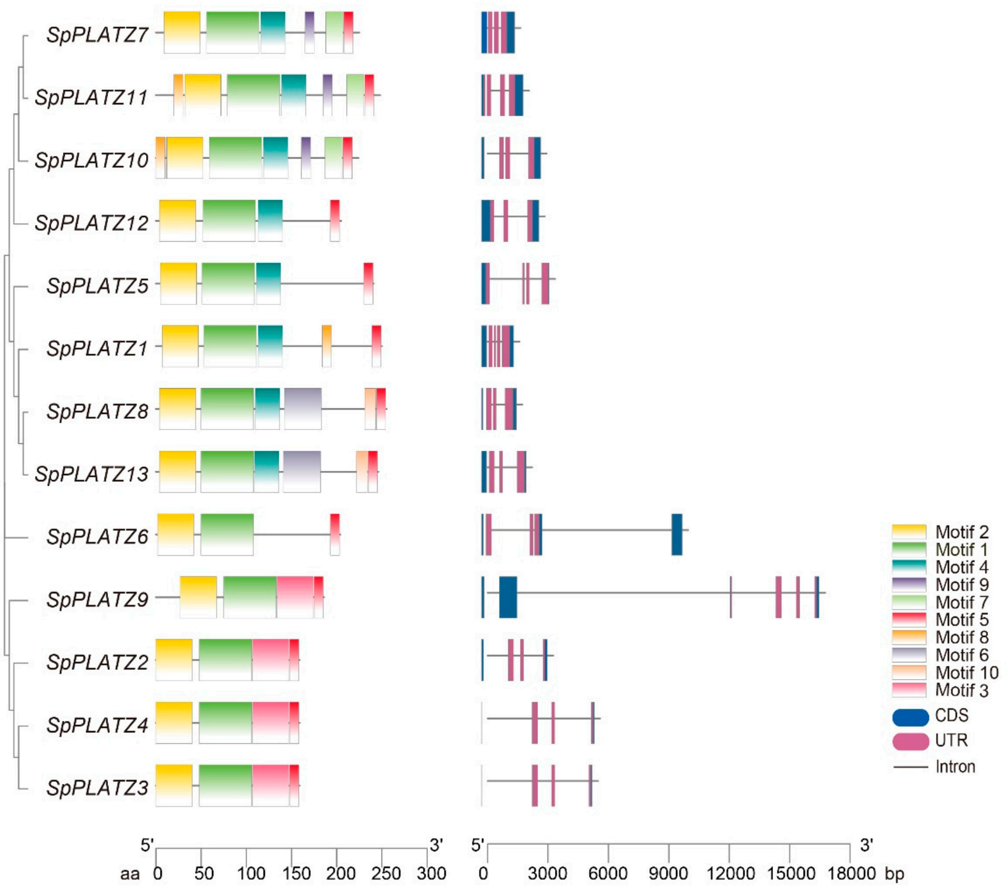

Figure S1. Analysis of motif structure, gene structure, and phylogenetic analysis of spPLATZ. (A) Ten amino acid motifs in the spPLATZ protein are indicated by a colored box. (B) The blue box indicates exons, the purple box indicates UTR, and the gray line indicates introns.

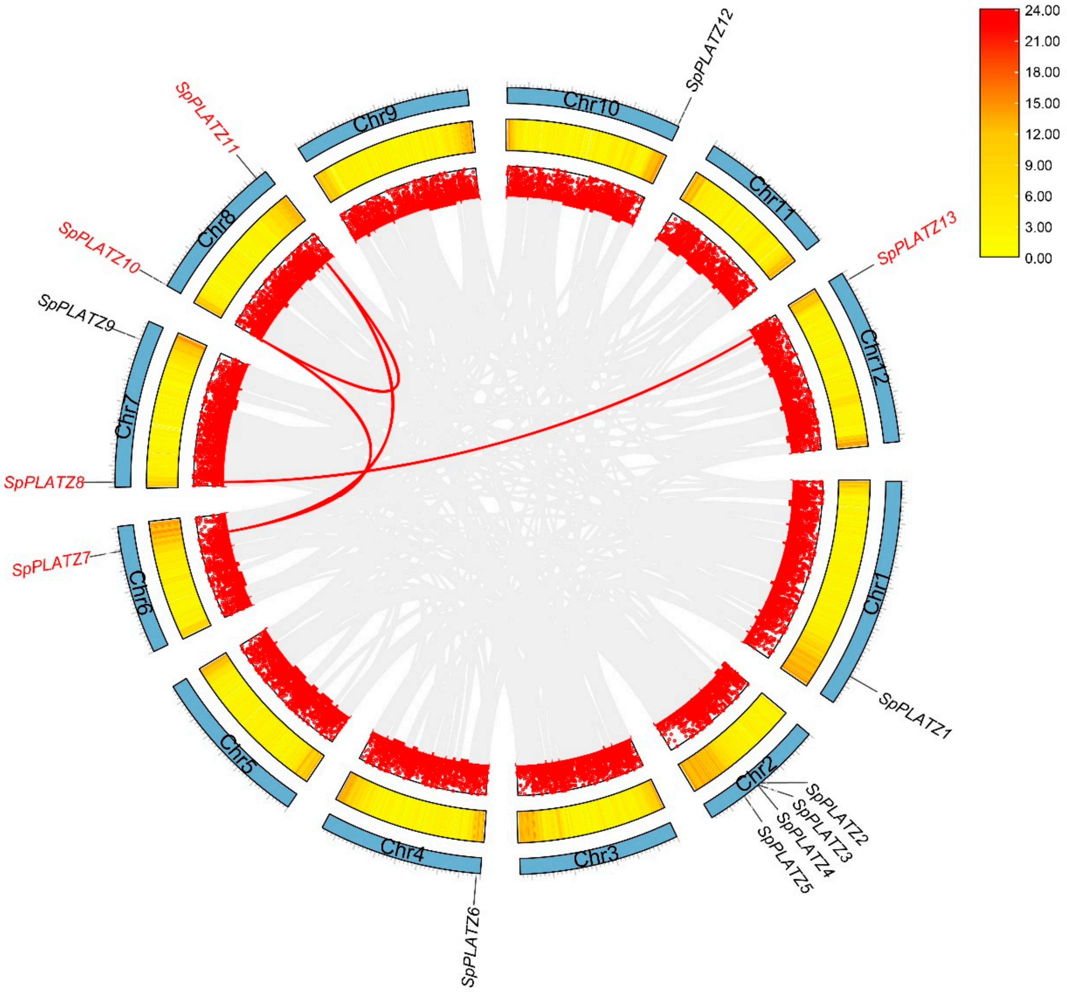

Figure S2. Gene duplication events in the genome, with the outer circle showing the chromosomes of wild tomato pennellii, the inner circle showing gene density, the red dot in the inner circle representing the N-ratio, and the ends of the lines representing direct homologous *spPLATZ* genes. The legend represents the values of the gene density, With red representing high levels and yellow representing low levels.

Table S2. The Ka/Ks ratios and date of duplication for duplicate SlPLATZ genes.

Duplicated Gene Pairs	Ka	Ks	Ka/Ks	Duplicated Type	Time (Mya*)
SlPLATZ18/SlPLATZ24	0.098	0.830	0.118	Segmental	27.667
SlPLATZ17/SlPLATZ21	0.095	0.798	0.119	Segmental	26.600
SlPLATZ21/SlPLATZ22	0.132	0.953	0.138	tandem	31.767

*Millions of years ago.

Table S3. The Ka/Ks ratios and date of duplication for duplicate S*pPLATZ* genes.

Duplicated Gene Pairs	Ka	Ks	Ka/Ks	Duplicated Type	Time (Mya*)
SpPLATZ7/SpPLATZ10	0.137	0.624	0.219	Segmental	20.813
SpPLATZ7/SpPLATZ11	0.098	0.757	0.129	Segmental	25.225
SpPLATZ8/SpPLATZ13	0.098	0.753	0.130	Segmental	25.103
SpPLATZ10/SpPLATZ11	0.127	0.881	0.144	tandem	29.376

*Millions of years ago.

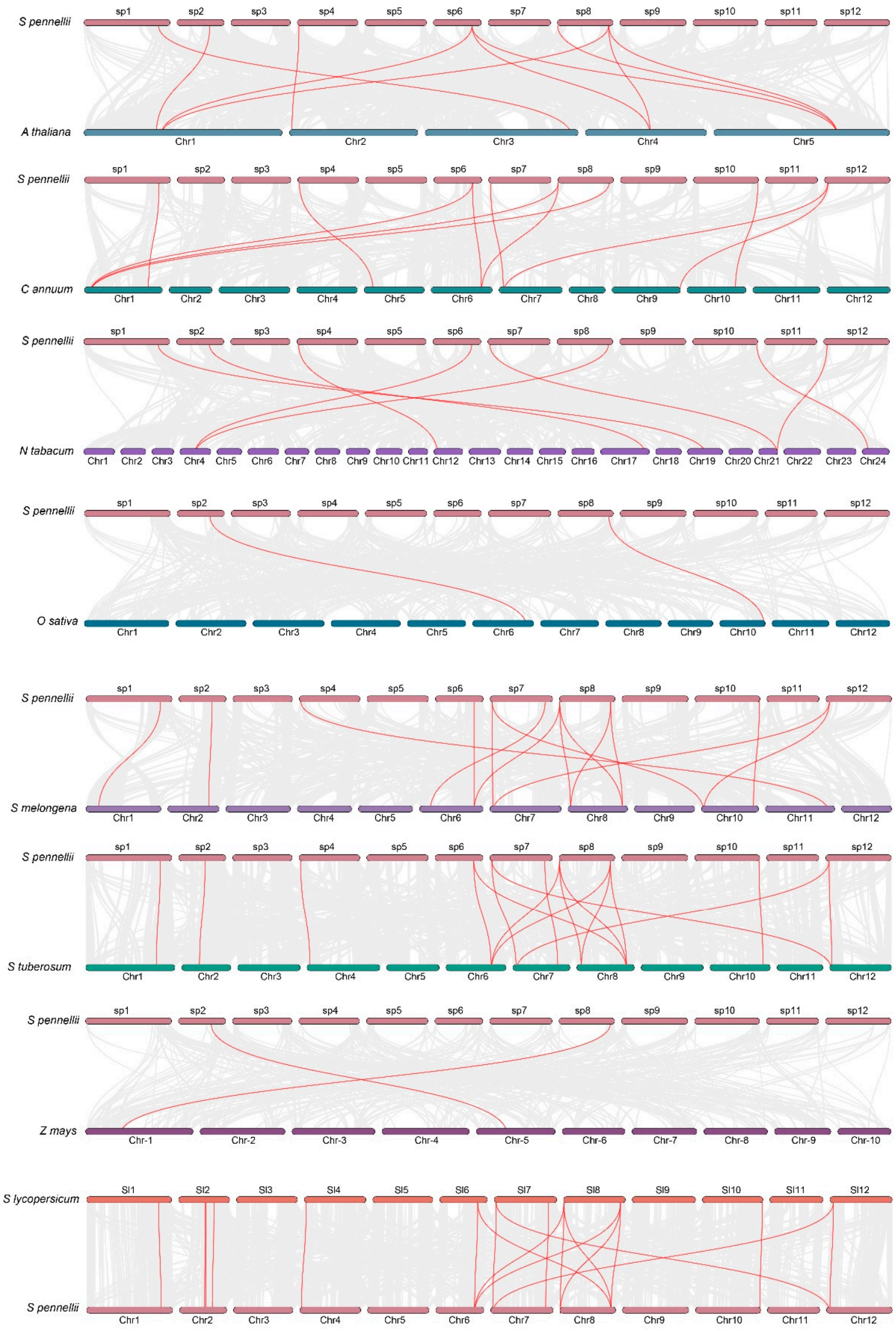

Figure S3. The collinearity diagram of *PLATZ* genes. Red lines highlight the homologous gene pairs of *spPLATZ* genes, and gray lines represent genome-wide collinear gene pairs.

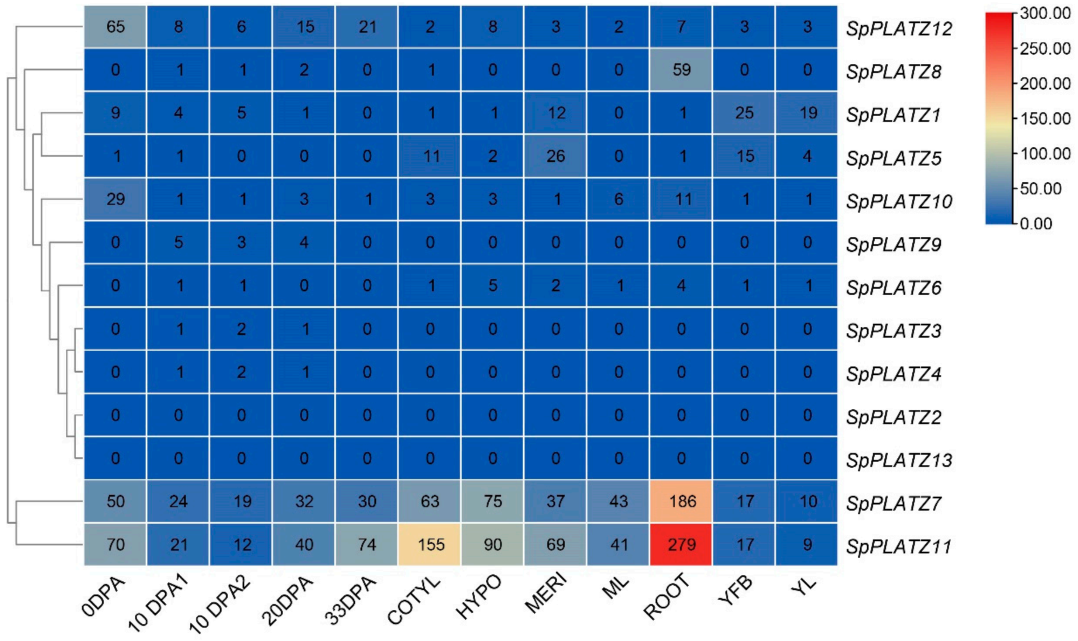

Figure S4. Heat map of tissue-specific expression of *PLATZ* genes in pennellii. The color bar represents the log2 expression values, with red representing high expression levels and blue representing low expression levels. The gene name is shown on the right side. Heinz 20 days post-anthesis fruit (20 DPA), Heinz 10 days post-anthesis fruit (10 DPA1), 10 days post-anthesis fruit 2 (10 DPA2), Heinz root (ROOT), Heinz anthesis flowers (0 DPA), Heinz vegetative meristems (MERI), Heinz young flower buds (YFB), Heinz young leaves (YL), Heinz hypocotyl (HYPO), cotyledons (COTYL), Heinz ripening fruit (33 DPA), and Heinz mature leaves (ML).
